# Ophthalmological findings in patients suspected with COVID-19 at a tertiary hospital in Pernambuco, Brazil

**DOI:** 10.5935/0004-2749.20220083

**Published:** 2025-02-11

**Authors:** Ana Karina Téles Silveira, Maria Isabel Lynch, Clistenes Stênio Lima De Medeiros, Bruno Teixeira De Moraes, Maria Cecília Remígio, Michelle Maria Figueiredo Paiva, Renata Ribeiro Silva, Virgínia Laura Lucas Torres

**Affiliations:** 1 Head and Neck Division, Ophthalmology Department, Hospital das Clínicas, Universidade Federal de Pernambuco, Recife, PE, Brazil; 2 Head and Neck Division, Otorhinolaryngology Department, Hospital das Clínicas, Universidade Federal de Pernambuco, Recife, PE, Brazil

**Keywords:** COVID-19, Coronavirus infections, SARS-CoV-2, Eye manifestations, Screening, COVID-19, Infecções por coronavírus, SARS-CoV-2, Manifestações oculares, Triagem

## Abstract

**Purpose:**

This study evaluated the frequency of the most common ophthalmological, neurological, and systemic findings in symptomatic patients seen at a COVID-19 screening service at Hospital das Clínicas - Universidade Federal de Per nambuco.

**Methods:**

A total of 104 patients under clinical suspicion of SARS-CoV-2 infection underwent medical evaluation through an ophthalmological and systemic symptoms survey. All participants selected for the study underwent COVID-19 RT-PCR testing.

**Results:**

The mean age was 38.8 years, with 44.23% between 31 and 40 years old, 68.27% female, and 31.73% male. The most common symptoms in patients with a positive RT-PCR test were cough (69.23%), fever (42.3%), hyposmia (38.46%), myalgia (38.46%), and ageusia (30.77%). In the positive group, 34.61% presented with ophthalmological symptoms: burning (19.23%), eye pain (11.54%), foreign body sensation (7.7%), hyperemia (7.7%), and tearing (3.84%).

**Conclusions:**

Systemic clinical features were characteristic of upper respiratory infection, but neurological findings of hyposmia and anosmia proved to be important markers for suspicion of SARS-CoV-2 infection. Ophthalmic symptoms in patients with COVID-19 were similar to those observed in other viral conditions and may precede systemic conditions. A high rate of self-medication was observed for general symptoms compared with ophthalmological conditions.

## Abstract (pt)

**Objetivo:**

Avaliar a frequência dos sintomas oftalmológicos, neurológicos e sistêmicos mais comuns em pacientes sintomáticos atendidos no serviço de triagem de COVID-19 do Hospital das Clínicas da UFPE.

**Métodos:**

Cento e quatro pacientes com suspeita clínica de infecção por Sars-Cov-2 foram submetidos a avaliação médica e aplicação de questionário sobre a sintomatologia oftalmológica, neurológica e sistêmica. Todos os participantes do estudo tiveram exame de RT-PCR para COVID-19 solicitado.

**Resultados:**

A média de idade foi de 38,8 anos, com 44,23% entre 31 e 40 anos. Mulheres corresponderam a 68,27% dos atendimentos e homens a 31,73%. Os sintomas mais frequentes nos pacientes com RT-PCR positivo foram: tosse (69,23%), febre (42,3%), mialgia (38,46%), hiposmia (38,46%), e ageusia (30,77%). Neste grupo, os sintomas oftalmológicos estiveram presentes em 34,61%, sendo: ardor (19,23%), dor ocular (11,54%), sensação de corpo estranho (7,7%), hiperemia (7,7%) e lacrimejamento (3,84%) os mais encontrados.

**Conclusões:**

O quadro clínico sistêmico foi característico de infecção respiratória alta, porém os achados neurológicos de hiposmia e anosmia mostraram-se importantes marcadores para a suspeição dos casos de infecção por COVID-19. Os sintomas oftalmológicos dos pacientes com COVID-19 foram semelhantes aos presentes em outros quadros virais, podendo preceder o quadro sistêmico. Houve uma alta taxa de automedicação para os sintomas gerais quando comparado ao quadro oftalmológico.

## INTRODUCTION

The new coronavirus pandemic started in the city of Wuhan, Hubei Province, China, on December 2019^(1)^ and reached Brazil around February 2020^(2)^. On August 18, 2020, Brazil had 3,407,354 confirmed cases and 109,888 deaths from the disease^(3)^.

Six types of coronavirus are related to disease in humans. Four types (229E, OC43, NL63, and HKU1) are widely distributed and cause flu-like symptoms^(4)^. The two other coronavirus types, severe acute respiratory syndrome coronavirus (SARS-CoV) and Middle East respiratory syndrome-related coronavirus (MERS- CoV) have a zoonotic origin and are related to fatal outcomes^(5)^. The latest coronavirus (SARS-CoV-2) is an enveloped, single-chained RNA beta-coronavirus that causes COVID-19^(1)^, a highly transmissible disease^(6)^. It is believed that the initial inoculation of SARS-CoV-2 occurred through contact with wild animals^(7)^.

Dissemination occurs through mucous membranes, whether oral, nasal, or ocular. Coughing, sneezing, and contact with contaminated objects can be a vehicle for the spread of viral particles^(8,9)^. The virus has been isolated by anal swab, suggesting fecal-oral transmission, as well as in urine^(10,11)^. Symptoms can appear from the second day and up to 14 days after infection^(12,13)^. People with comorbidities and elderly people are more likely to develop severe illness^(14)^.

As it is a new disease, the evolution, signs, and symptoms of COVID-19 are not fully known, but the most frequent findings are cough, fever, and dyspnea^(14)^. Less specific symptoms that have been documented include runny nose, sore throat, diarrhea, asthenia, anosmia/hyposmia, ageusia, headache, mental confusion, chest pain, nausea, vomiting, myalgia, eye pain, and urticaria^(14-16)^.

Complications include pneumonia, kidney failure, cardiomyopathy, encephalopathy, and stroke. X-ray ground-glass opacities interstitial pneumonia, also found in other viral pneumonias, appear in cases with greater severity^(15)^.

Ocular manifestations caused by coronavirus such as uveitis, retinitis, vasculitis and optic neuritis can be severe in animals^(17)^. However, manifestations in humans are generally mild and rare. No description of ocular manifestations have been reported in the literature in other epidemics caused by other coronaviruses^(18,19)^, despite the fact that SARS-CoV was isolated from tears of SARS patients^(20)^. Other types of coronavirus can cause conjunctivitis in humans, for example human coronavirus NL63 (HCoV-NL63), first described in a baby with bronchiolitis and conjunctivitis^(21)^ and subsequently in 28 children, of whom 17% had conjunctivitis^(22)^.

The literature describes the presence of SARS-CoV-2 in tears^(23)^, as well as findings of mild follicular conjunctivitis in patients with COVID-19, with signs such as conjunctival hyperemia, chemosis, secretion, and epiphora. These findings are more frequent in critically ill patients who require hospitalization but may occur as the first sign of infection^(24,25)^.

The differential diagnosis includes other viral conjunctivitis, bacterial or allergic conjunctivitis, herpes simplex keratitis, anterior uveitis, corneal abrasion, foreign body, dry eye syndrome, exposure keratopathy in intubated patients, and chemosis in critically ill patients^(26)^. Ophthalmologists may therefore be the first to suspect SARS-CoV-2 infection since conjunctivitis may be one of the symptoms of the disease. In fact, a Chinese ophthalmologist, Dr. Li Wenliang, who ended up dying of COVID-19 after possible transmission by an asymptomatic patient with glaucoma, was one of the first to warn of an emerging virus^(27)^. A case series by Wu et al. on 38 patients with COVID-19 in Hubei Province reported 12 patients showing ocular symptoms compatible with conjunctivitis, four of which had moderate disease, two with severe disease, and six in critical condition^(24)^.

Hospital das Clínicas-Universidade Federal de Pernambuco (UFPE), a tertiary university hospital, became a COVID-19 reference center in the State of Pernambuco, Brazil, on April 18, 2020. A screening service for patients with COVID-19-related symptoms started at this time and ophthalmologists, otorhinolaryngologists, and psychiatrists were enlisted in the team.

To date, no study has described ophthalmological findings in patients symptomatic of COVID-19 in Brazil. Because it is a new, poorly understood health issue, the description of ophthalmological and neurological findings in symptomatic patients, as well as the relationship between these findings and viral PCR results, may bring awareness regarding the condition, facilitating diagnosis and prevention of transmission.

## METHODS

The present study adopted a cross-sectional design and was approved by the Research Ethics Committee of Hospital das Clínicas-UFPE under protocol 4.073.278.

Physicians used a questionnaire (Appendix 1) to assess patients seen at the COVID-19 screening service at Hospital das Clínicas-UFPE who had flu-like symptoms or a clinical suspicion of SARS-CoV-2 infection.

All patients were requested to undergo a SARS-CoV-2 RT-PCR test according to the protocol established by the State Health Department. The results of these tests were recorded along with the symptoms reported by the patients. Participants were divided into two groups, positive and negative RT-PCR, to compare the systemic and ophthalmological symptoms. Patients were asked to sign an informed consent form (Appendix 2) indicating their voluntary participation in the research.

For statistical analysis, simple percentages and means and standard deviation were used to express data and calculated using the IBM SSPS Statistics^®^ software.

## RESULTS

Between May and August 2020, 104 patients seen at the screening service for COVID-19 at Hospital das Clínicas-UFPE, were submitted to the questionnaire. The mean age of the patients was 38.8 years, and 44.23% were in the 31 to 40 years age group (Table 1). The majority of patients were female (68.27%), and 36.54% held an undergraduate degree.

**Table 1:** Epidemiological data

Variable	Subgroups	Total patients
General		104
Age group	<20 years	0
	21-30 years	20 (19.23%)
	31-40 years	46 (44.23%)
	41-50 years	21 (20.19%)
	51-60 years	17 (16.34%)
	>70 years	0
Gender	Male	33 (31.73%)
	Female	71 (68.27%)
Education level	Middle School	7 (6.73%)
	High School	27 (25.96%)
	Undergraduate degree	38 (36.54%)
	Graduate school	9 (8.65%)
	Uninformed	23 (22.11%)
Laboratory examination for	Swab (RT-PCR)	79 (75.96%)
COVID-19	Not performed	25 (24.04%)
Swab Result (RT-PCR)	Positive	26 (32.91%)
	Negative	52 (65.82%)
	Inconclusive	1 (1.26%)

Most patients went to the hospital at the early onset of symptoms, and 50.00% were seen by the 3rd day of symptom onset. Out of 104 participants, 79 (75.96%) underwent COVID-19 RT-PCR test (Table 1). Twenty-six (32,91%) individuals tested positive, and these participants, twelve (46.15%) were seen between the 4th and 7^th^ day of symptom onset.

The most frequent symptoms presented by the patients were cough (54.8%), asthenia (37.5%), headache (34.61%), and fever (30.77%) (Figure 2). Ophthalmological symptoms were observed in 33.65% of the participants.

The most prevalent symptoms in patients positive on COVID-19 RT-PCR were cough (69.23%), fever (42.3%), myalgia (38.46%) hyposmia (38.46%), and ageusia (30.77%). The prevalence of ophthalmic symptoms was 34.61%, which included burning (19.23%), pain (11.54%), foreign body sensation (7.7%), hyperemia (7.7%), and tearing (3.84%). In this group, the ophthalmic clinical picture started concomitantly with general symptoms in 77.77% of the cases. In one patient (11.11%), symptoms appeared one day before flu-like symptoms, and after 3 days in another (11.11%), began after three days.

**Figure 1:**
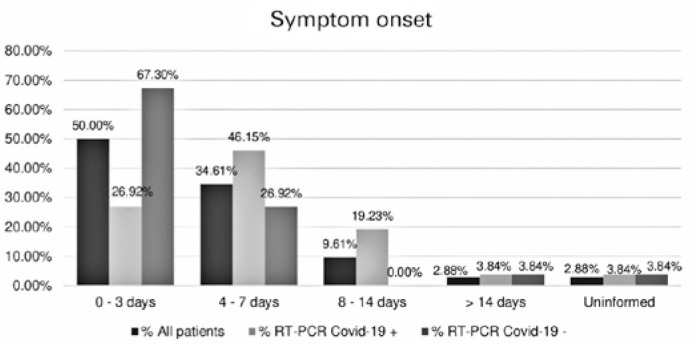
Time until symptom onset.

**Figure 2:**
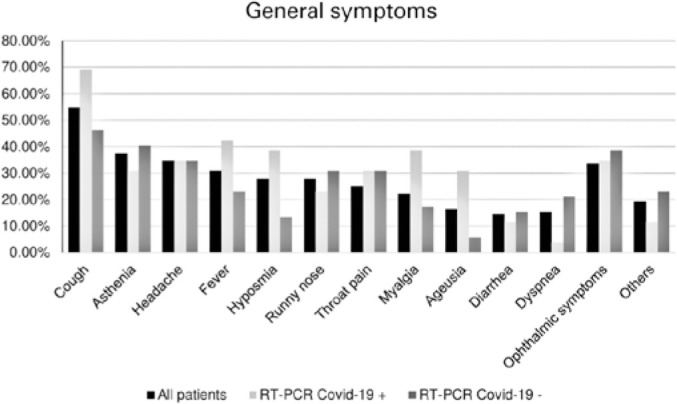
Symptoms reported.

**Figure 3:**
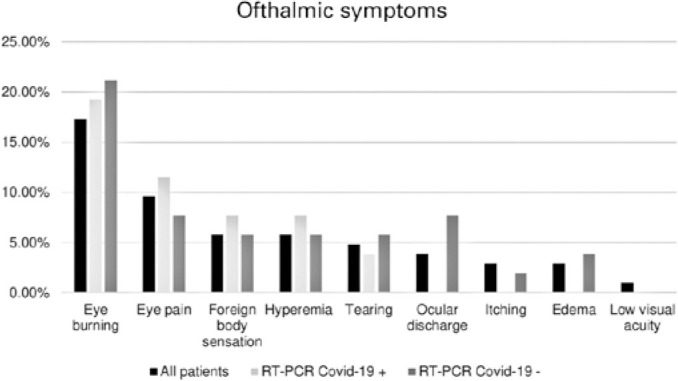
Ophthalmic symptoms reported.

Patients with negative RT-PCR results most frequently presented cough (46.15%), asthenia (40.38%), headache (34.61%), runny nose (30.77%), and throat pain (30.77%). Ophthalmological symptoms were present in 38.46% of cases in this group (Figure 3), including burning (21.15%), pain (7.7%), ocular discharge (7.7%), foreign body sensation (5.77%), hyperemia (5.77%), and tearing (5.77%). For this group, ocular symptoms started 

around the same time as general symptoms for 80% of cases.

For systemic symptoms, 44 patients reported selfmedication (42.3% of 104) with analgesics/antipyretics (68.18% of 44 individuals), azithromycin (29.54%), ivermectin (27.27%), and nitazoxanide (4.54%). Regarding eye complaints, two of the 35 patients who presented symptoms (5.71%) used eye drops for self-medication (ketorolac trometamol and carmellose sodium). Of the patients enrolled, 58 (55.77%) did not undergo any type of treatment, either for general or ocular symptoms, without medical advice.

## DISCUSSION

A high level of education of most participants, as well as the mean age of the sample, including the absence of elderly people, can be explained by the fact that a significant number of them were employees of Hospital das Clínicas-UFPE or their relatives.

Systemic symptoms of COVID-19 found in the present study were similar to those found in other epidemiological analyses^(12,14,15)^ but with a lower frequency, probably because they were treated at a screening service, in which patients were seen at the beginning of the clinical picture. That is, 73.07% of the patients who tested positive were seen within 7 days of the onset of the first symptom. Although patients presented symptoms similar to other respiratory infections, the neurological findings of hyposmia and ageusia proved to be important markers in the differentiation and suspicion of cases of SARS-CoV-2 infection.

Awareness of the existence of other manifestations of the disease, as described in the present research, is important. Ophthalmological symptoms were present in 34.61% of the patients with laboratory confirmation for COVID-19 and started concomitantly with the other systemic symptoms in 78% of the cases. Interestingly, in 11% of those diagnosed with COVID-19, the ocular symptoms anticipated the systemic ones. These findings are important especially because they can be early indicators of the disease and could possibly refer to alternative ways of transmission apart from the respiratory route. Similar findings were also observed in a study of 535 patients in Wuhan, China^(28)^, which, in addition to clinical signs such as dry eye and conjunctival injection, reported symptoms of ocular discharge (9.7%), foreign body sensation (11, 77%), tearing (10.28%), itching (9.9%), photophobia (2.99%), and visual blurring (12.71%)^(28)^. In a case report published in November 2020, Marquezan et al. describe a patient with severe conjunctivitis: yellowish conjunctival discharge, foreign body sensation, redness, and tearing^(29)^. In a study published in June 2020, Valente et al.^(30)^ reported that four out of 27 (15%) hospitalized pediatric patients in Rome, Italy, had ocular symptoms compatible with mild viral conjunctivitis.

Regarding neurological symptoms (hyposmia and ageusia), the positive RT-PCR group had a substantially higher prevalence (38.46% with hyposmia and 30.77% with ageusia) than those with negative RT-PCR results (13.46% with hyposmia and 5.77% with ageusia). This observation is important for clinical suspicion of oligosymptomatic flu cases, which thus expediates the isolation and monitoring of these patients.

In the present analysis, a low self-medication rate for ophthalmic symptoms was observed compared with that for systemic manifestations. Only two of the 35 (5.71%) participants used medication on their own for ocular conditions, a substantially lower percentage than the 42.3% who self-medicated for the treatment of systemic conditions. This could be due to either the mildness of ocular symptoms or lack of public knowledge about the treatment of ophthalmic disease.

The study was limited by the relatively small sample size. However, the study findings present evidence that COVID-19 features ophthalmological manifestations, which may present earlier than the respiratory and neurological symptoms commonly reported in COVID-19 studies across the world. Describing These descriptions are necessary in speeding up the diagnosis, as well as provide reference for the investigation of possible alternative disease transmission routes.
